# Supplementary material for: A synthetic cell-free 36-enzyme reaction system for vitamin B12 production
Source: Nat Commun. 2023 Aug 24;14:5177. doi: 10.1038/s41467-023-40932-4 (PMC10449867; doi:10.1038/s41467-023-40932-4)
Supplement: Supplementary file 3 — Supplementary Information [file 41467_2023_40932_MOESM3_ESM.pdf]

SUPPLEMENTARY INFORMATION

A synthetic cell-free 36-enzyme reaction system for vitamin B_{12} production

Qian Kang[1,2,3], Huan Fang [1,2,3], Mengjie Xiang[1,2], Kaixing Xiao[2,3], Pingtao Jiang [2,3], Chun You[1,2], Sang Yup Lee[4*], Dawei Zhang[1,2,3*]

[1]University of Chinese Academy of Sciences, No.19 (A) Yuquan Road, Shijingshan District, Beijing 100049, China.

[2]Tianjin Institute of Industrial Biotechnology, Chinese Academy of Science, 32 Xi Qi Dao, Tianjin Airport Economic Area, Tianjin 300308, China.

[3]Key Laboratory of Engineering Biology for Low-Carbon Manufacturing, Tianjin Institute of Industrial Biotechnology, Chinese Academy of Science, Tianjin 200208, China.

[4]Department of Chemical and Biomolecular Engineering (BK21 four program), Korea Advanced Institute of Science and Technology (KAIST), 291 Daehak-ro, Yuseong-gu, Daejeon, 34141, Republic of Korea.

* For correspondence:
Sang Yup Lee, leesy@kaist.ac.kr

Dawei Zhang, Zhang_dw@tib.cas.cn

Supplementary Table 1
Supplementary Table 2
Supplementary Figure 1-14
Source Data file: Supplementray Figure 9b

Supplementary Table 1: Information of enzymes used in the enzymatic platform.

Enzyme Abbr.	Full Name	EC Number	Source Organism	ΔG'° (kcal/mol) [a]	K'eq
HemB	Porphobilinogen synthase	4.2.1.24	*Sinorhizobium meliloti* 320	-24.7	1.4×10^{18}
	2 5-Aminolevulinate → Porphobilinogen + 2 H_2O				
HemC	Porphobilinogen deaminase	2.5.1.61	*Sinorhizobium meliloti* 320	-28.3	6.0×10^{20}
	4 Porphobilinogen + H_2O → Hydroxymethylbilane + 4 Ammonia				
HemD	Uroporphyrinogen III synthase	4.2.1.75	*Sinorhizobium meliloti* 320	-13.2	-
	Hydroxymethylbilane → Uroporphyrinogen III + H_2O				
CobA	Uroporphyrin-III $^{(C2,7)}$ -methyltransferase	2.1.1.107	*Rhosobacter capsulatus* SB1003	-20.5	-
	2 SAM + Uroporphyrinogen III → 2 SAH + Precorrin-2				
CobI	Uroporphyrin-III $^{(C20)}$ - methyltransferase	2.1.1.130	*Rhosobacter capsulatus* SB1003	-19.8	-
	Precorrin-2 + SAM → Precorrin-3A + SAH				
CobG	Precorrin 3B synthase	1.14.13.83	*Brucella melitensis* bv. 1 str. 16M	-105.7	-
	Precorrin-3A + NADH → Precorrin-3B + NAD^+				
CobJ	Precorrin 3B $^{(C17)}$ - methyltransferase	2.1.1.131	*Rhosobacter capsulatus* SB1003	-66.3	-
	Precorrin-3B+ SAM → Preccorrin-4 +SAH				
CobM	Precorrin 4 $^{(C11)}$ - methyltransferase	2.1.1.133	*Rhosobacter capsulatus* SB1003	-43.6	-
	Precorrin-4 + SAM → Precorrin-5 + SAH				
CobF	Precorrin 5 $^{(C1)}$ - methyltransferase	2.1.1.152	*Rhosobacter capsulatus* SB1003	-32.3	-

	Precorrin-5 + SAM $\rightarrow$ Precorrin-6X + SAH + Ace				
CobK	Precorrin-6A reductase	1.3.1.54	*Rhosobacter capsulatus* SB1003	17.9	-
	Precorrin-6X + NADPH $\rightarrow$ Precorrin-6Y + $NADP^+$				
CobL	Precorrin 6B $^{(C5, 15)}$ -methyltransferase	2.1.1.132	*Rhosobacter capsulatus* SB1003	-3.2	-
	Precorrin-6Y + 2 SAM $\rightarrow$ Precorrin-8 + 2 SAH				
CobH	Precorrin-8X methylmutase	5.4.99.61	*Rhosobacter capsulatus* SB1003	49.2	-
	Precorrin-8 $\rightarrow$ Hydrogenobyrinate				
CobB	Hydrogenobyrinate *a, c*-diamide synthease	6.3.5.9	*Rhosobacter capsulatus* SB1003	-0.6	-
	Hydrogenobyrinate + 2 ATP + 2 L-Gln $\rightarrow$ Hydrogenobyrinate *a, c*- diamide + 2 ADP + 2 L-Glu				
CobN	Cobaltochelatase	6.6.1.2	*Brucella melitensis* bv. 1 str. 16M	8.0	-
CobS	Cobaltochelatase	6.6.1.2	*Sinorhizobium meliloti* 320		
CobT	Cobaltochelatase	6.6.1.2	*Sinorhizobium meliloti* 320		
	Hydrogenobyrinate *a, c*- diamide + ATP + Co^{2+} $\rightarrow$ Cob(II)yrinate a, c-diamide + ADP				
CobR	Cob (II)yrinate *a, c*-diamide reductase	1.16.8.1	*Brucella melitensis* bv. 1 str. 16M	-10.2	-
	Cob(II)yrinate *a, c*-diamide + 0.5 $FMNH_2$ $\rightarrow$ Cob(I)yrinate *a, c*-diamide + FMN				
Fre	NADH-flavin reductase	1.5.1.41	*Eschericia coli* MG1655	-36.1	-
	FMN + NADH $\rightarrow$ $FMNH_2$ + NAD^+				

CobA	Cobinamide adenosyltransferase	2.5.1.17	*Salmonella typhimurium* AR2680	59.0	-
	Cob(I)yrinate *a, c*-diamide + ATP → Adenosylcobyrinate *a, c*-diamide + triphosphate				
CbiP	Adenosylcobyric acid synthase	6.3.5.10	*Sinorhizobium meliloti* 320	-8.6	-
	Adenosylcobyrinate *a, c*-diamide + 4 ATP + 4 L-Gln → Adenosylcobyrate + 4 ADP + 4 L-Glu				
PduX	L-threonine kinase	2.7.1.177	*Salmonella typhimurium* AR2680	-16.2	-
	L-threonine + ATP → L- Threonine 3-O-P + ADP				
CobC	Threonine-phosphate decarboxylase	4.1.1.81	*Sinorhizobium meliloti* 320	-3.1	1.8×10^{2}
	L- Threonine 3-O-P + H^{+} → (R)-1-Amino-2-propanol-O-2-P				
CbiB	Adenosylcobinamide-phosphate synthase	6.3.1.10	*Propionibacterium freudenreichii* ATCC 9614	-7.1	-
	Adenosylcobyrate + (R)-1-Amino-2-propanol-O-2-P + ATP → Adenosylcobinamide-P + ADP				
CobU	Cobinamide phosphate guanylyltransferase	2.7.7.62	*Eschericia coli* MG1655	-103.3	-
	Adenosylcobinamide-P + GTP → Adenosylcobinamide-GDP + diphosphate				
CobT	Nicotinate-nucleotide-dimethybenzimidazole phosphoribosyltransferase	2.4.2.21	*Eschericia coli* MG1655	-6.3	4.1×10^{4}
	5, 6 -dimethylbenzimidazole + NMN → α- ribazole-5'-P + Nicotinate				
CobS	Adenosylcobalamin-5'-phosphate synthase	2.7.8.26	*Eschericia coli* MG1655	2.7	-

	Adenosylcobinamide-GDP +α- ribazole-5'-P → Adenosylcobinamide-5-P + GMP				
CobC	Adenosylcobalamin 5'-phosphate phosphatase	3.1.3.73	*Eschericia coli* MG1655	-3.7	5.0×10^{2}
	Adenosylcobinamide-5-P →Adenosylcobalamin + Pi				
HemA	5-aminolevulinate synthase	2.3.1.37	*Rhodobacter sphaeroides* 2.4.1	4.5	4.7×10^{-4}
	Succinyl-CoA + Glycine → 5-ALA + CoA				
RocG	NAD-glutamate dehydrogenase	1.4.1.2	*Bacillus subtilis* 168	7.6	2.5×10^{-6}
	L-glutamate + NAD^+ → 2-oxoglutarate + NADH				
GlnA	Glutamine synthetase	6.3.1.2	*Eschericia coli* MG1655	-4.1	1.0×10^{3}
	L-glutamate + ATP → L-glutamine + ADP				
OGDH Lpd	Lipoamide dehyfrogenase	3.4.24.59	*Eschericia coli* MG1655		
OGDH SucA	Subunit of E1(0) component of 2-oxoglutarate dehydrogenase	1.2.4.2	*Eschericia coli* MG1655	-7	1.4×10^{5}
OGDH SucB	dihydrolipoyltranssuccinylase	1.8.1.4	*Eschericia coli* MG1655		
	2-oxoglutarate + NAD^+ + CoA → Succinyl-CoA + NADH				
PpK	Polyphosphate kinase	2.7.4.1	*Corynebacterium glutamicum* ATCC 13032	-	-
	ADP + $Pi_{(n)}$ → ATP + $Pi_{(n-1)}$				
PpA	Inorganic pyrophosphatase	3.6.1.1	*Eschericia coli* MG1655	4.1<br>-108.9[b]	-
	Triphosphate + H_2O → Diphosphate + Phosphate<br>Diphosphate + H_2O → 2 Phosphate				

[a] The standard Gibbs free energy change ($\Delta G'^{o}$) of HemB, HemC, CobC (*Sinorhizobium meliloti* 320), CobT, CobC (*Eschericia coli* MG1655), HemA, RocG,

GlnA, OGDH (Lpd SucA SucB) enzymatic reaction at pH 8.0 and ionic strength of 0.1 M (http://equilibrator.weizmann.ac.il/). Another enzymatic reaction at pH 7.3 and ionic strength of 0.25 M (https://metacyc.org/).

[b] PpA catalyzed decomposition of diphosphate into phosphate, $\Delta G'^{o}$= 4.1 kcal/mol; PpA catalyzed decomposition of triphosphate into diphosphate and phosphate, $\Delta G'^{o}$= -108.9 kcal/mol

Supplementary Table 2. Components of different AdoCbl synthetic system in Figure 8.

Reaction		5-ALA →AdoCbl								HBA →AdoCbl			
	Group ID	1	2	3	4	5	6	7	8	1	2[f]	3	4
Enzymes (μM)	HemB	0.1	0.1	0.1	0.1	0.1	0.32	6.76	6.76				
	HemC	1	1	1	1	1	2.27	1.52	1.52				
	HemD	1	1	1	1	1	2.12	2	2				
	CobA [1]	10	10	10	10	10	10	10	10				
	Usage of HBA CCE (OD_{600})/ 250μl reactant	5	10	10	10	10	30	30	30				
	TP of HBA CCE (mg/ml)	3.1	6.2	6.2	6.2	6.2	18.7	18.7	18.7				
	MetK							10	10				
	MtnN							10	10				
	CobB	1	3	3	3	3	3	3	3	3	3	3	3
	CobN	5	10	10	10	10	10	15	15	5	15	15	15
	CobST	5	10	10	10	10	10	10	10	10	10	10	10
	CobR	3	3	3	3	3	3	90	90	30	180	180	90
	Fre	3	3	3	3	3	3	9	9	9	9	18	9
	CobA[2]	3	3	3	3	3	3	3	3	10	30	60	30
	CbiP	3	3	3	3	3	3	9	9	30	45	180	90
	CobC[3]	0.5	10	10	10	10	10[a]	20[a]	20[a]	20	10[a]	30[a]	30[a]
	PduX	0.5	10	10	10	10	10[a]	20[a]	20[a]	20	10[a]	30[a]	30[a]
	CobU	3	3	3	3	3	3	50	50	30	80	95.24	95.24
	CobT	3	3	3	3	3	3[b]	20[b]	20[b]	20	60[b]	52.64[b]	52.64[b]
	CobC[4]	3	3	3	3	3	3	100	100	30	100	83.33	83.33
	CobS	20	20										
	CbiB	20	20										
	SIMPLExCobS			20	20	20	20	20	20	20	20	20	20
	SIMPLExCbiB			20	20	20	20	20	20	20	20	20	20
Substrate and cofactors	5-ALA (mM)	2.5	5	1	1	1	5	3	3				
	HBA (μM)									10	25	40	40
	SAM (mM)	1	1	1	1	1	1						
	L-Met (mM)							2	2				
	L-glutamine (mM)	2	2	1	1	1	1	3	3	3	3	5	5
	FMN (mM)	1	1	0.5	0.5	0.5	0.5	0.5	0.5	0.5	0.1	0.1	0.1
	NADH (mM)	3	5	1	1	1	5	5	5	5	5	5	5
	NADPH (mM)	1	2	1	1	1	5	5	5			2	2
	L-threonine (mM)	0.5	0.5	1	1	1	5[a]	5[a]	5[a]	5	5[a]	5[a]	5[a]
	PLP (mM)	0.5	0.5	0.5	0.5	0.5	0.5[a]	3[a]	3[a]	3	1[a]	3[a]	3[a]
	DMBI (mM)	0.25	0.25	1	1	1	5[b]	5[b]	5[b]	5	5[b]	5[b]	5[b]

	NMN (mM)	0.25	0.25	1	1	1	5[b]	5[b]	5[b]	5	5[b]	5[b]	5[b]
	GTP (mM)	0.5	0.5	0.5	0.5	0.5	2	2	2	1	1	1	1
	BSA (mg/ml)	2.5	2.5	2.5	2.5	2.5	1	1	1	1	1	1	1
	PpK (μM)	0.5			1	3	3	45	45	10	10	10	10
	PpA (μM)	3	3	3	3	3	3	90	90	30	90	90	90
	RocG (μM)	3	3	3	3	3	3	3	3	10			
	ODGH (μM)				3	3	3		10				
Regeneration module	HemA (μM)				3	3	3		5				
	GlnA (μM)				10	10	10						
	Glycine (mM)				5	5	5		2				
	SMPP (mM)	5			5	5	5	10	10	15	20	20	20
	CoA (mM)				0.1	0.1	0.1		0.1				
Buffers		100 mM Tris-HCl (pH 8.0)					100 mM Hepes-NaOH (pH8.0)			100 mM MOPS-NaOH (pH8.0)			
In batches		-[c]	-[c]	-[c]	-[c]	-[c]	+[d]	+[d]	+[d]	-[c]	+[e]	+[e]	+[e]
Final volume (ml)		2	4	2	4	2	0.5	0.25	0.25	5	0.25	1	1

[a] Threonine consumption branch pathway was incubated in 15 mM Hepes buffer (pH 7.4) for 5 h in corresponding concentrations before added in AdoCbl synthetic system.

[b] CobT reaction was incubated in 50 mM Glycine-NaOH buffer (pH 10.0) for 5 h in corresponding concentrations before added in AdoCbl synthetic system.

[c] One-pot reaction.

[d] Two-pots reaction contained 5-ALA to HBA and HBA to AdoCbl.

[e] Two-pots reaction contained HBA to CBAD and CBAD to AdoCbl.

[f] Enzymes applied in this group were used after freshly purified immediately.

[1] Uroporphyrin-III$^{(C2,7)}$-methyltransferase, CobA, from *R. capsulatus*.

[2] Cobinamide adenosyltransferase, CobA, from *S. typhimurium*.

[3] Threonine-phosphate decarboxylase, CobC, from *S. meliloti*.

[4] Adenosylcobalamin-5'-phosphate synthase, CobC, from *E. coli*.

Supplementary figures and figure legends

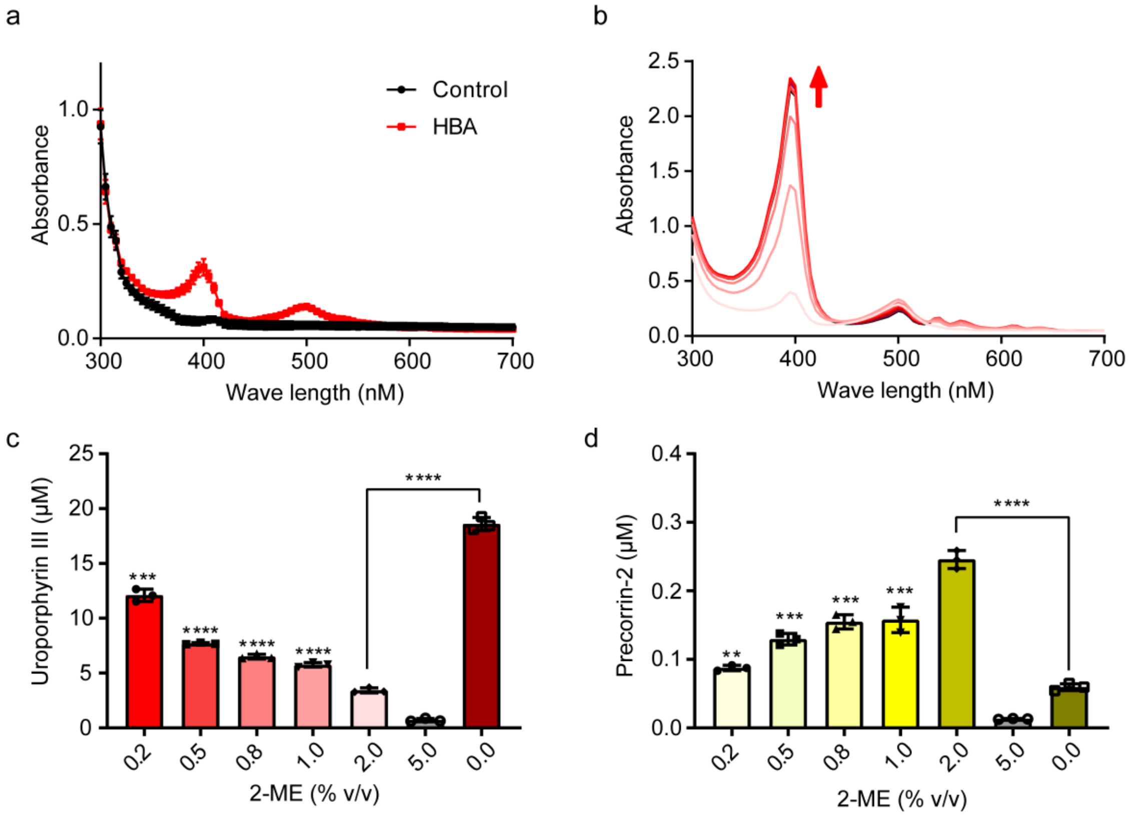

Supplementary Figure 1. Discovery and solution of the accumulated dead-end by-product uroporphyrin III in the precursor module.

a, Absorption spectrum of the initial HBA reactant. HBA: 0.1 μM HemB, 1 μM HemC, 1 μM HemD, 10 μM CobA, 1 μM CobI, 1 μM CobG, 1 μM CobJ, 1 μM CobM, 1 μM CobF, 1 μM CobK, 1 μM CobL, 1 μM CobH, 5 mM 5-ALA, 5 mM SAM, 2 mM NADH, 1 mM NADPH, 5 mM $MgCl_2$, 10 mM KCl, 5 mM NaCl in 50 mM Tris-HCl (pH 8.0) buffer; Control: HBA reactant described above without addition of 5-ALA and SAM. Reaction was performed in 32°C for 12 h and terminated with boiling water bath for 30 min, samples were detected using microplate reader. Reactions were performed in duplicate (n=2 biologically independent samples) and shown with line running through the mean values ± SD. b, Spontaneous oxidation of uroporphyrinogen III. Absorption spectrum of uroporphyrinogen III standards was monitored every hour exposed to air for 7 hours. c and d, The titration of β-mercaptoethanol (2-ME) was performed in the precursor module. The addition of 2% (v/v) 2-ME led to a significant decrease in the oxidation of uroporphyrinogen III and an increase in the production of precorrin-2.

Reactions were performed in triplicate (n=3 biologically independent samples) and data are presented as mean values ± SD. Two-sided unpaired t test is carried out with different 2-ME addition reaction with no 2-ME addition reaction. Unpaired t test in Supplementary Figure 1c: 0.2 to 0.0, P =0.0002 (t=13.7); 0.5 to 0.0, P <0.0001 (t=31.41); 0.8 to 0.0, P <0.0001 (t=32.9); 1.0 to 0.0, P <0.0001 (t=35.24); 2.0 to 0.0, P <0.0001 (t=41.11); 5.0 to 0.0, P <0.0001 (t=50.41). Unpaired t test in Supplementary Figure 1d: 0.2 to 0.0, P =0.0017 (t=7.469); 0.5 to 0.0, P =0.0002 (t=12.47); 0.8 to 0.0, P =0.0001 (t=14.32); 1.0 to 0.0, P =0.0009 (t=8.876); 2.0 to 0.0, P <0.0001 (t=22.89); 5.0 to 0.0, P <0.0001 (t=15.77). **, P < 0.01; ***, P < 0.001; ***, P < 0.0001. Source data are provided as a Source Data file.

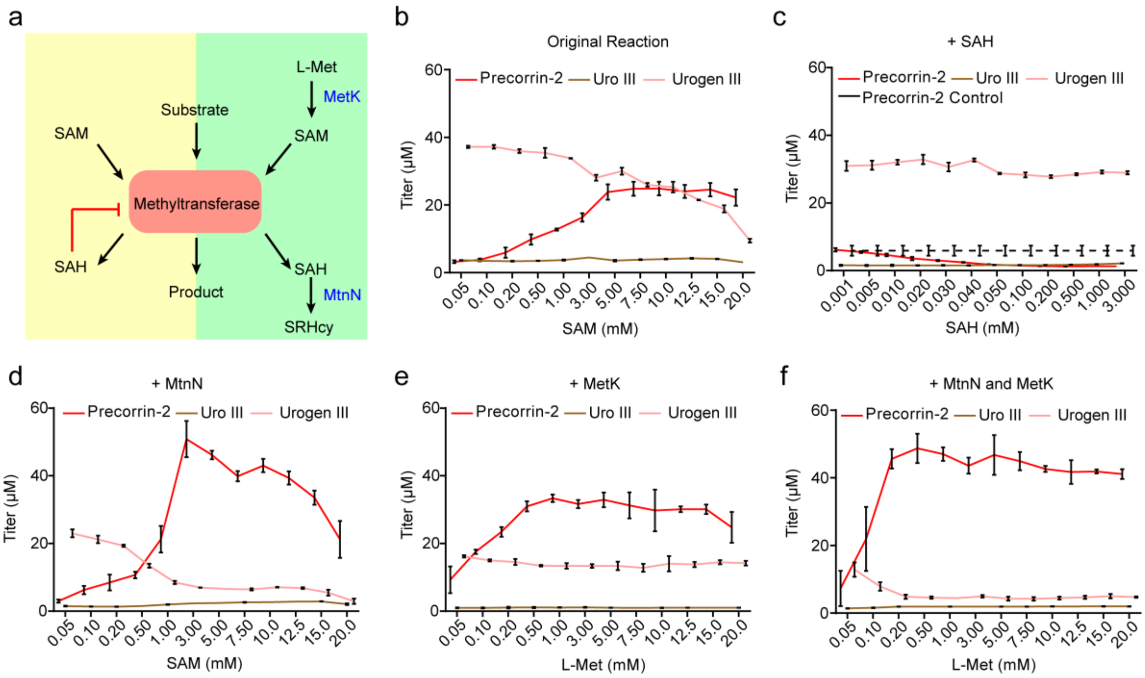

Supplementary Figure 2. Relieving SAH feedback inhibition to methyltransferases in the precursor module.

a, Schematic diagram of methyl transformation in our reaction system. The left yellow part shows the initial catalysis pathway, methyltransferase transfers a methyl from SAM to the substrate, leaving SAH. SAH feed-back inhibited methyltransferase. The right green part is the optimized catalysis pathway, in which MetK catalyzes L-Met into SAM, while SAH is transformed into S-ribosyl homocysteine (SRHcy) by MtnN. b, Titration of SAM in precursor module reaction with 0.1 μM HemB, 1 μM HemC, 1 μM HemD, 10 μM CobA and 5 mM 5-ALA in 50 mM Tris-HCl (pH 8.0) buffer. c, Titration of SAH in precursor module reaction with 0.1 μM HemB, 1 μM HemC, 1 μM HemD, 10 μM CobA, 5 mM 5-ALA and 0.2 mM SAM in 50 mM Tris-HCl (pH 8.0) buffer. Precorrin-2 Control with dotted line is a baseline of precorrin-2 titer without SAH addition. d, Titration of SAM in precursor module reaction with 0.1 μM HemB, 1 μM HemC, 1 μM HemD, 10 μM CobA, 5 μM MtnN and 5 mM 5-ALA in 50 mM Tris-HCl (pH 8.0) buffer. e, Titration of L-Met in precursor module reaction with 0.1 μM HemB, 1 μM HemC, 1 μM HemD, 10 μM CobA, 5 μM MetK, 5 μM PpK, 2 mM AMP, and 1 mM SMPP and 5 mM 5-ALA in 50 mM Tris-HCl (pH 8.0) buffer. f, Titration of L-Met in precursor module reaction with 0.1 μM HemB, 1 μM HemC, 1 μM HemD, 10 μM

CobA, 5 μM MtnN, 5 μM MetK, 5 μM PpK, 2 mM AMP, and 1 mM SMPP and 5 mM 5-ALA in 50 mM Tris-HCl (pH 8.0) buffer. Reaction above were incubated in 32℃ for 2 h, then treated and detected according to which described in the detection methods. In supplementary Figure 2b to 2f, all reactions were performed in triplicate (n=3 biologically independent samples) and shown with line running through the mean values ± SD. Abbreviation: Uro III, uroporphyrin III; Urogen III, uroporphyrinogen III; SAM, S-adenosyl-methionine; SAH, S-adenosyl-homocysteine; SRHcy, S-ribosyl homocysteine; L-Met, L-methionine. Source data are provided as a Source Data file.

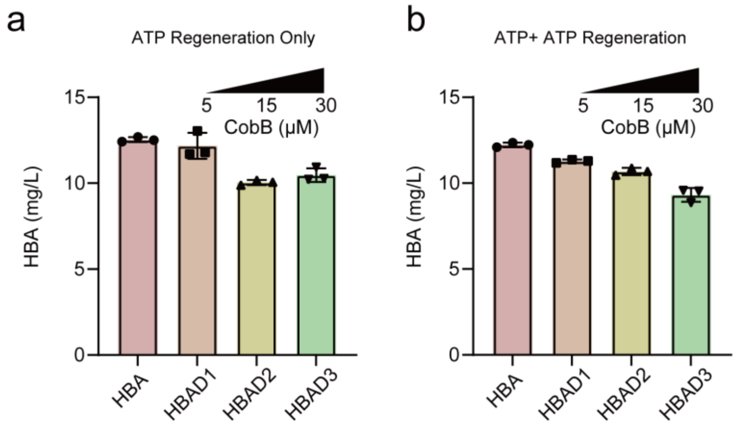

Supplementary Figure 3 Driving force of CobB reaction to HBA titer *in vitro*.

a, driving force of CobB reaction to HBA synthesis using ATP regeneration only. HBA: 0.1 μM HemB, 1 μM HemC, 1 μM HemD, 10 μM CobA, 5 μM MetK, 5 μM PpK, 10 μM MtnN, 2 mM AMP, 1 mM SMPP, 5 mM 5-ALA, 1 mM L-Met, 0.236 mM NADH, 0.236 mM NADPH, 2 mM $MgCl_2$, 10 mM KCl, 5 mM NaCl, 155.8 mg/ml wet cell weight of HBA CCE (corresponding 17.81 mg/ml dry cell weight, 6.23 mg/ml total protein, and 10 OD_{600} HBA cell culture fluid per volume reactant) in 50 mM Tris-HCl (pH 8.0) buffer. HBAD1: HBA reactant with 5 μM CobB and 1 mM L-glutamine; HBAD2: HBA reactant with 10 μM CobB and 1 mM L-glutamine; HBAD3: HBA reactant with 15 μM CobB and 1 mM L-glutamine. Reactions were performed in triplicate (n=3 biologically independent samples) and data are presented as mean values ± SD. b. driving force of CobB reaction to HBA synthesis using ATP and ATP regeneration both. HBA: 0.1 μM HemB, 1 μM HemC, 1 μM HemD, 10 μM CobA, 5 μM MetK, 5 μM PpK, 10 μM MtnN, 2 mM ATP, 1 mM SMPP, 5 mM 5-ALA, 1 mM L-Met, 0.236 mM NADH, 0.236 mM NADPH, 2 mM $MgCl_2$, 10 mM KCl, 5 mM NaCl, 155.8 mg/ml wet cell weight of HBA CCE (corresponding 17.81 mg/ml dry cell weight, 6.23 mg/ml total protein, and 10 OD_{600} HBA cell culture fluid per volume reactant) in 50 mM Tris-HCl (pH 8.0) buffer. HBAD1: HBA reactant with 5 μM CobB and 1 mM L-glutamine; HBAD2: HBA reactant with 10 μM CobB and 1 mM L-glutamine; HBAD3: HBA reactant with 15 μM CobB and 1 mM L-glutamine. Reactions were performed in triplicate (n=3 biologically independent samples) and data are presented as mean values ± SD. Source data are provided as a Source Data file.

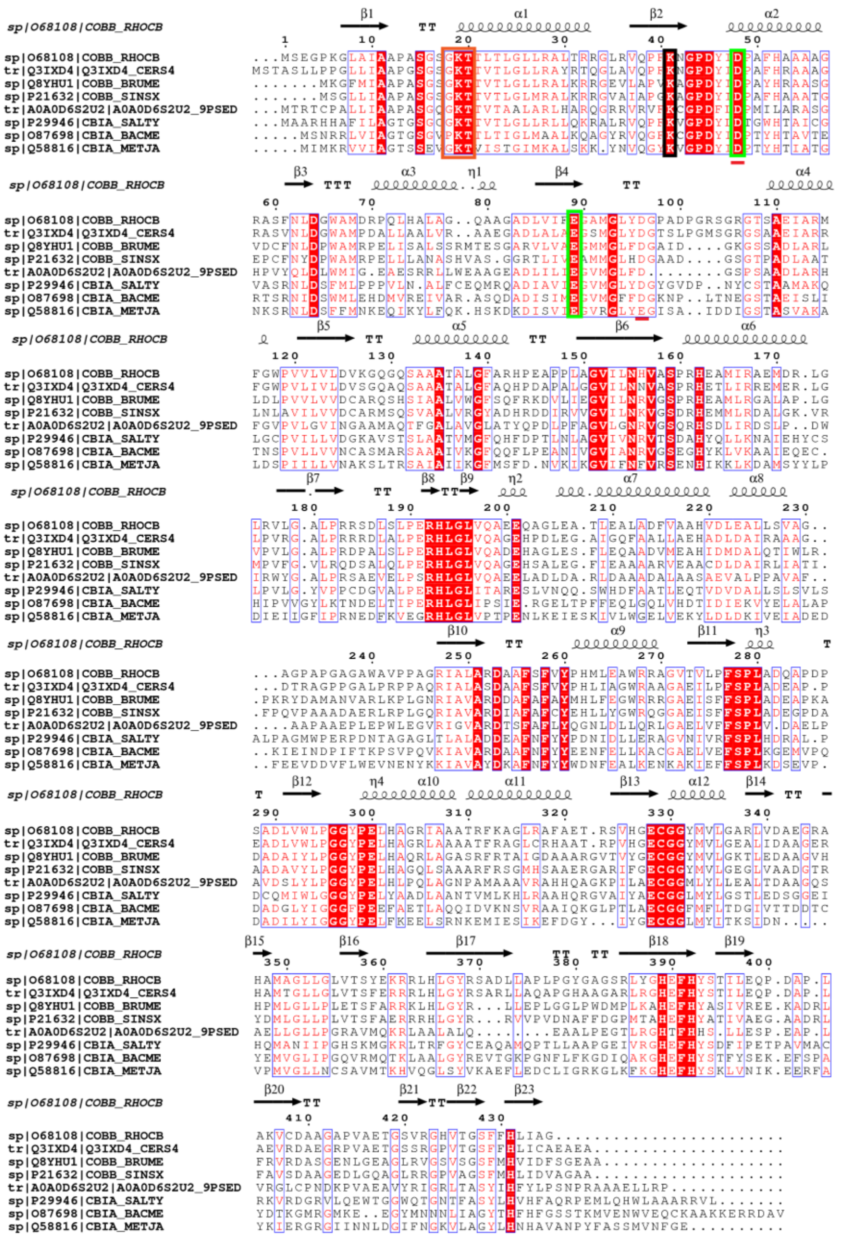

Supplementary Figure 4. Sequence alignment of hydrogenobyrinate *a, c*-diamine synthases from eight different organisms.

The ATP-binding residues are shown in the orange box, the active catalytic residues are in the black box, the Mg^{2+}-binding residues are in the green box, and predicted substrate binding residues are underlined in red. All protein sequence were acquired from UniProt (https://www.uniprot.org/)[1] , and referenced structure of COBB_RHOCB (https://alphafold.ebi.ac.uk/search/text/COBB_RHOCB) was acquired from AlphaFold Protein Structure Database (https://alphafold.ebi.ac.uk/)[2, 3]. The sequence alignment was performed using CLUSTALW (https://www.genome.jp/tools-bin/clustalw) and ESPript 3.0 (https://espript.ibcp.fr/ESPript/ESPript/index.php)[4] . COBB_RHOCB

(https://www.uniprot.org/uniprotkb/O68108/entry) is CobB from *R. capsulatus*; Q3IXD4_CERS4 (https://www.uniprot.org/uniprotkb/Q3IXD4/entry) is CobB from *R. sphaeroides*; COBB_BRUME (https://www.uniprot.org/uniprotkb/Q8YHU1/entry) is CobB from *B. melitensis*; COBB_SINSX (https://www.uniprot.org/uniprotkb/P21632/entry) is CobB from *S. meliloti.*; A0A0D6S2U2_9PSED (https://www.uniprot.org/uniprotkb/A0A0D6S2U2/entry) is *P. denitrificans*; CBIA_SALTY (https://www.uniprot.org/uniprotkb/P29946/entry) is CbiA from *S. typhimurium*; CBIA_BACME (https://www.uniprot.org/uniprotkb/O87698/entry) is CbiA from *B. megaterium*; CBIA_METJA is CbiA from *M. jannaschii*.

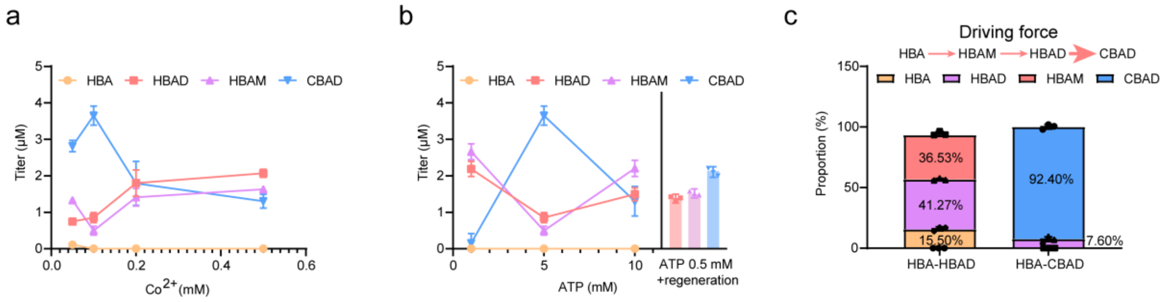

Supplementary Figure 5. Strengthen the CobB-CobNST cascade reaction.

a, Titration of Co^{2+} in the CobB-CobNST reaction, containing 5 μM HBA, 3 μM CobB, 5 μM CobN, 5 μM CobST, 2 mM L-glutamine, 5 mM ATP in MOPS buffer (pH 8.0) with 5 mM $MgCl_2$, 10 mM KCl and 5 mM NaCl. The indicated amount of $CoCl_2$ was added. Reactions were performed in triplicate (n=3 biologically independent samples) and shown with line running through the mean values ± SD. b, Titration of ATP in the CobB-CobNST reaction, containing 5 μM HBA, 3 μM CobB, 5 μM CobN, 5 μM CobST, 2 mM L-glutamine, 0.1 mM $CoCl_2$ in MOPS buffer (pH 8.0) with 5 mM $MgCl_2$, 10 mM KCl and 5 mM NaCl. ATP was added as indicated, and the 0.5+ reaction additionally contained 3 μM PpK, 1 mM AMP and 5 mM SMPP. Reactions were performed in triplicate (n=3 biologically independent samples) and shown with line running through the mean values ± SD. c. Driving force of the cascade reaction of CobB and CobNST. HBA-HBAD: 10 μM HBA, 3 μM CobB, 3 μM PpK, 2 mM L-glutamine, 10 mM AMP in MOPS buffer (pH 8.0) with 5 mM $MgCl_2$, 10 mM KCl and 5 mM NaCl; HBA-CBAD: 10 μM HBA, 3 μM CobB, 15 μM CobN, 10 μM CobST, 3 μM PpK, 2 mM L-glutamine, 0.2 mM $CoCl_2$, 10 mM AMP in MOPS buffer (pH 8.0) with 5 mM $MgCl_2$, 10 mM KCl and 5 mM NaCl. Reactions above were performed in 32°C for 2 h, then treated and detected according to which described in the detection method. Reactions were performed in triplicate (n=3 biologically independent samples) and data are presented as mean values ± SD. Source data are provided as a Source Data file.

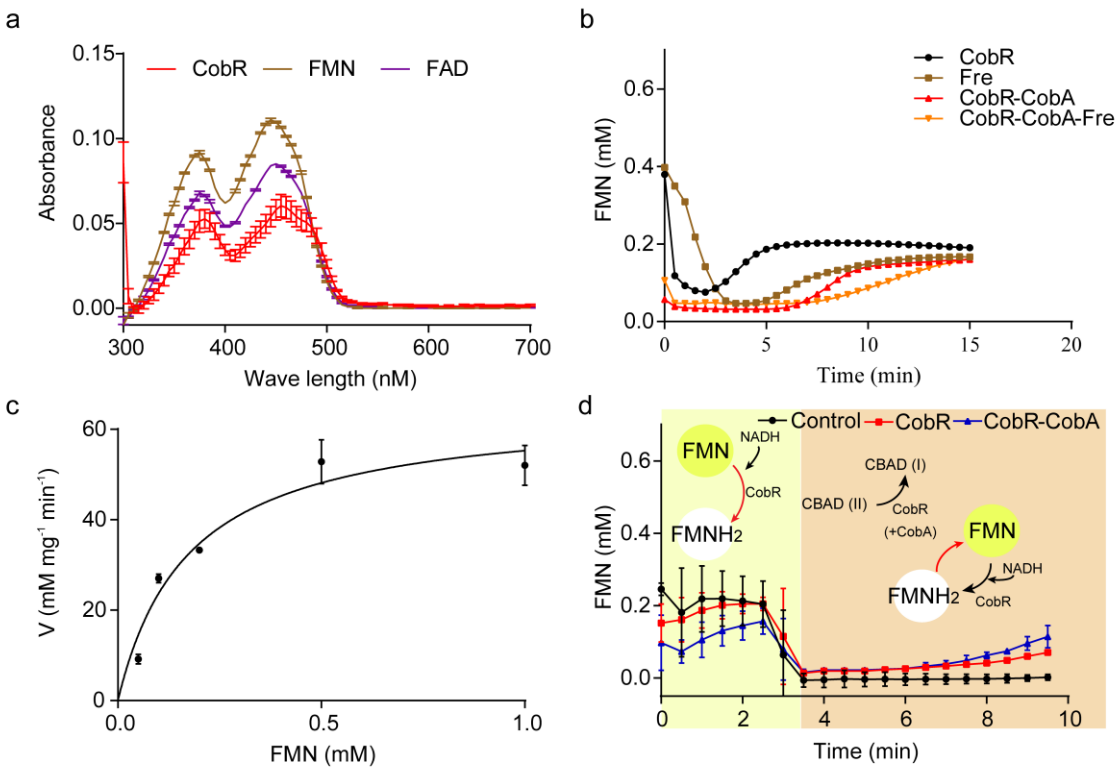

Supplementary Figure 6. Enzymatic catalysis of CobR.

a, Absorption spectrum of purified *Bm*CobR. The spectral curve of *Bm*CobR has two peaks at 380 nm and 455 nm, The spectral curve of standard FMN has two peaks at 375 nm and 450 nm, The spectral curve of standard FAD has two peaks at 375 nm and 450 nm. All data are shown from n=3 independent biological samples with line running through the mean values ± SD. b. NADH-dependent FMN reductase activity of *Bm*CobR. Redox state of FMN were continuously monitored over 15 min at 32℃. CobR: 0.5 mM FMN, 5 mM NADH and 5 μM CobR; Fre: 0.5 mM FMN, 5 mM NADH and 5 μM Fre; CobR-CobA: 0.5 mM FMN, 5 mM NADH, 5 μM CobR and 5 μM CobA; CobR-CobA-Fre: 0.5 mM FMN, 5 mM NADH, 5 μM CobR 5 μM CobA and 5 μM Fre. c. Enzyme kinetic parameters of *Bm*CobR for the reduction of FMN. Reactions were performed in triplicate (n=3 biologically independent samples) and shown with line running through the mean values ± SD. d. CBAD reduction catalyzed by CobR. CobR: FMN, NADH and CobR were incubated at 32℃ for 3 min to generate $FMNH_2$, and the CBAD reactant was added for the next 6 min of reaction; Control: same as CobR group but CBAD reactant was replaced by fCBAD (fake CBAD, CBAD reaction without HBA input); CobR-CobA: same as CobR group but CobA was added to the initial of

reaction. the FMN concentration was monitored every 30 s during the entire reaction. Reactions were performed in duplicate (n=2 biologically independent samples) and shown with line running through the mean values ± SD. Source data are provided as a Source Data file.

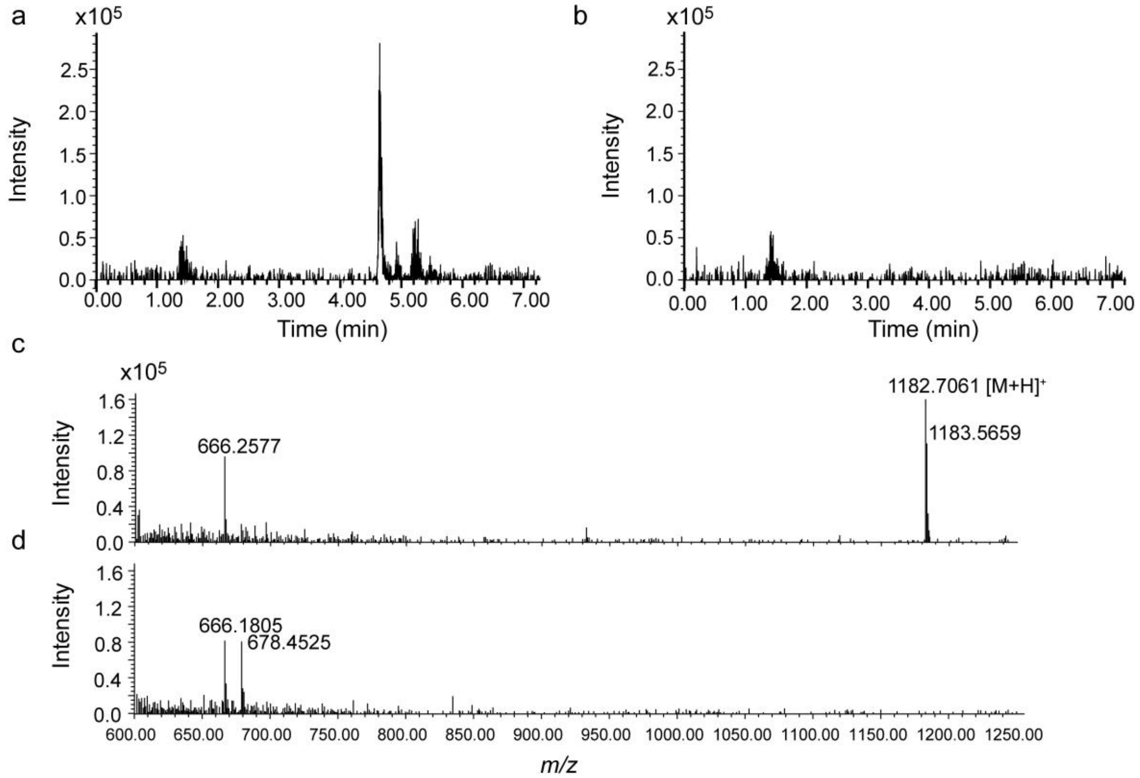

Supplementary Figure 7. Photolysis of synthetic AdoCby.

a, MS signal extract of the synthetic AdoCby sample. b. MS signal extract of the AdoCby sample irradiated with 280 lux of light at 455 nm for 1 hour. c. MS detection of AdoCby. d. MS detection of AdoCby after photolysis. Source data are provided as a Source Data file.

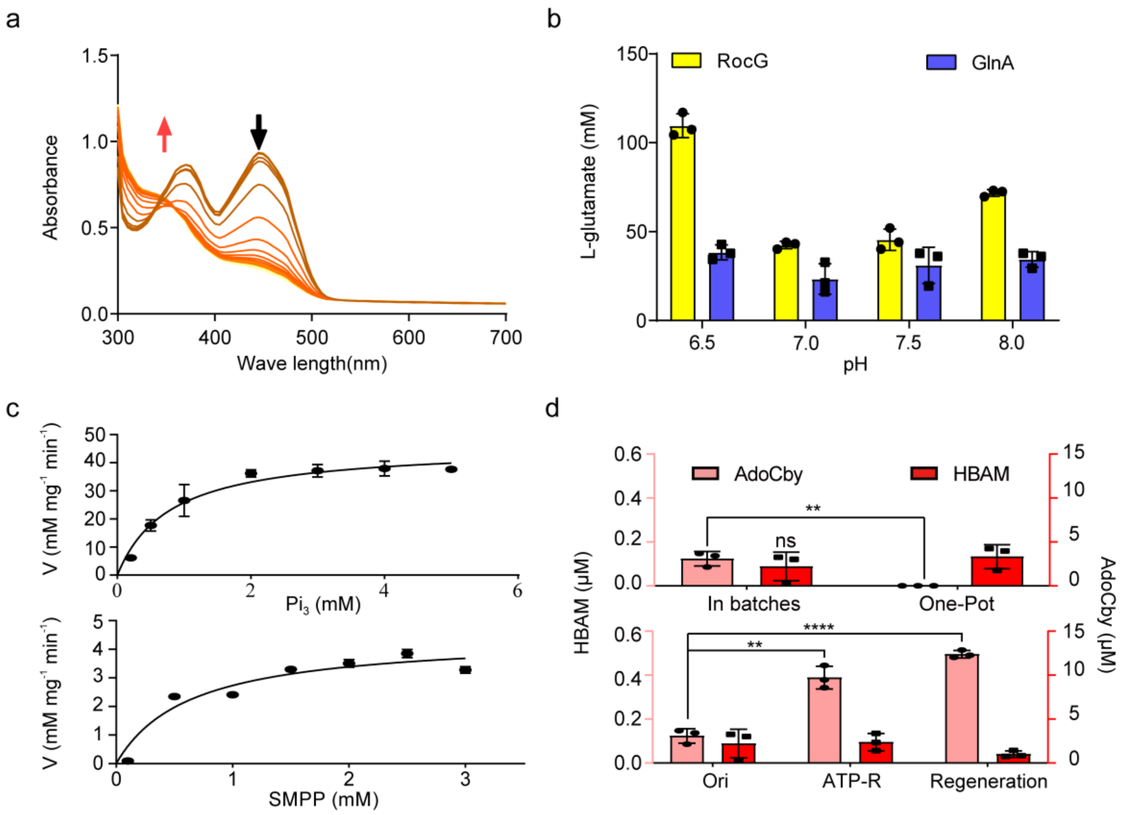

Supplementary Figure 8. Enzymological characteristics and application of the regeneration systems.

a, RocG regenerated NADH from NAD^+ in CobR reaction. After CobR catalyzed the reduction of FMN for 15 min, the formed $FMNH_2$ was oxidized in air and the spectrum of the reactant is shown in dark brown line. When 10 µM RocG and 5 mM L-glutamate were added, the spectral changes were monitored every minute for 20 min. In this process, the absorption at 340 nm (NADH) increased and the absorption at 450 nm (FMN) decreased showing that RocG reduced NAD^+ to NADH, promoting the reduction of FMN by CobR. The color change from brown to yellow corresponds to the passage of time and the changes of spectrum were monitored every minute. b, L-glutamate consumption by RocG and GlnA in buffers with different pH values. Here, 2 mM L-glutamate, 1 mM NAD^+, 1 mM NH_4Cl, 10 µM RocG and 10 µM GlnA are incubated in 100 mM Hepes-NaOH buffer with 5 mM NaCl, 50 mM KCl, 50 mM NaCl and different pH values ranging from 6.5 to 8.0 for 20 min. The consumption of L-glutamate was calculated by measuring the concentrations of NADH and phosphate. Reactions were performed in triplicate (n=3 biologically independent samples) and data

are presented as mean values ± SD. c. Enzyme kinetic parameters of PpA with triphosphate and PpK of SMPP. Reactions were performed in triplicate (n=3 biologically independent samples) and data are presented as mean values ± SD. d. optimization of AdoCby synthetic module. upper subgraph: In batches: 10 μM HBA, 3 μM CobB, 15 μM CobN, 10 μM CobST, 2 mM L-Gln, 10 mM ATP, 0.2 mM $CoCl_2$, was incubated in 100 mM MOPS buffer with 5 mM $MgCl_2$, 10 mM KCl and 5 mM NaCl at 32°C for 2 h, then the reactant was concentrated by vacuum condenser. The concentrated reactant was then incubated with 18 μM CobR, 6 μM CobA, 9 μM Fre, 90 μM PpA, 18 μM CbiP, 2 mM NADPH, 5 mM NADH, 2 mM L-Gln, 5 mM ATP, 0.8 mM $MnCl_2$ at 32°C for 12 h. One-pot: 10 μM HBA, 3 μM CobB, 15 μM CobN, 10 μM CobST, 0.2 mM $CoCl_2$, 18 μM CobR, 6 μM CobA, 9 μM Fre, 90 μM PpA, 18 μM CbiP, 2 mM NADPH, 5 mM NADH, 4 mM L-Gln, 15 mM ATP, 0.8 mM $MnCl_2$ at 32°C for 14 h. Reactions were performed in triplicate (n=3 biologically independent samples) and data are presented as mean values ± SD. Two-sided unpaired t test is carried out with the titer of HBAM and AdoCby between in batches reaction and one-pot reaction. Unpaired t test: HBAM, P=0.4140 (t=0.9106); AdoCby, P=0.0029 (t=6.486). ns, P> 0.05; **, P < 0.01. lower subgraph: Ori: 10 μM HBA, 3 μM CobB, 15 μM CobN, 10 μM CobST, 18 μM CobR, 6 μM CobA, 9 μM Fre, 90 μM PpA, 18 μM CbiP, 2 mM L-glutamine, 5 mM ATP, 0.8 mM $MnCl_2$, 0.2 mM $CoCl_2$, 5 mM $MgCl_2$, 10 mM KCl in 100 mM MOPS buffer (pH 8.0) were incubated in anaerobic chamber in 32°C kept away from light for 12 h; ATP-R: Ori group with 30 μM PpK and 10 mM SMPP; Regeneration: Ori group with 30 μM PpK, 30 μM GlnA, 30 μM RocG and 10 mM SMPP. Reactions were performed in triplicate (n=3 biologically independent samples) and data are presented as mean values ± SD. Two-sided unpaired T test is carried out with the titer of HBAM and AdoCby between the ATP-R reaction or Regeneration reaction with Ori reaction. Unpaired t test of HBA titer: ATP-R to Ori, P=0.8878 (t=0.1503); Regeneration to Ori, P=0.2788 (t=1.252). Unpaired t test of AdoCby titer: ATP-R to Ori, P=0.0017 (t=7.509); Regeneration to Ori, P<0.0001 (t=17.38). ns, P> 0.05; **, P < 0.01; ****, P < 0.0001. Source data are provided as a Source Data file.

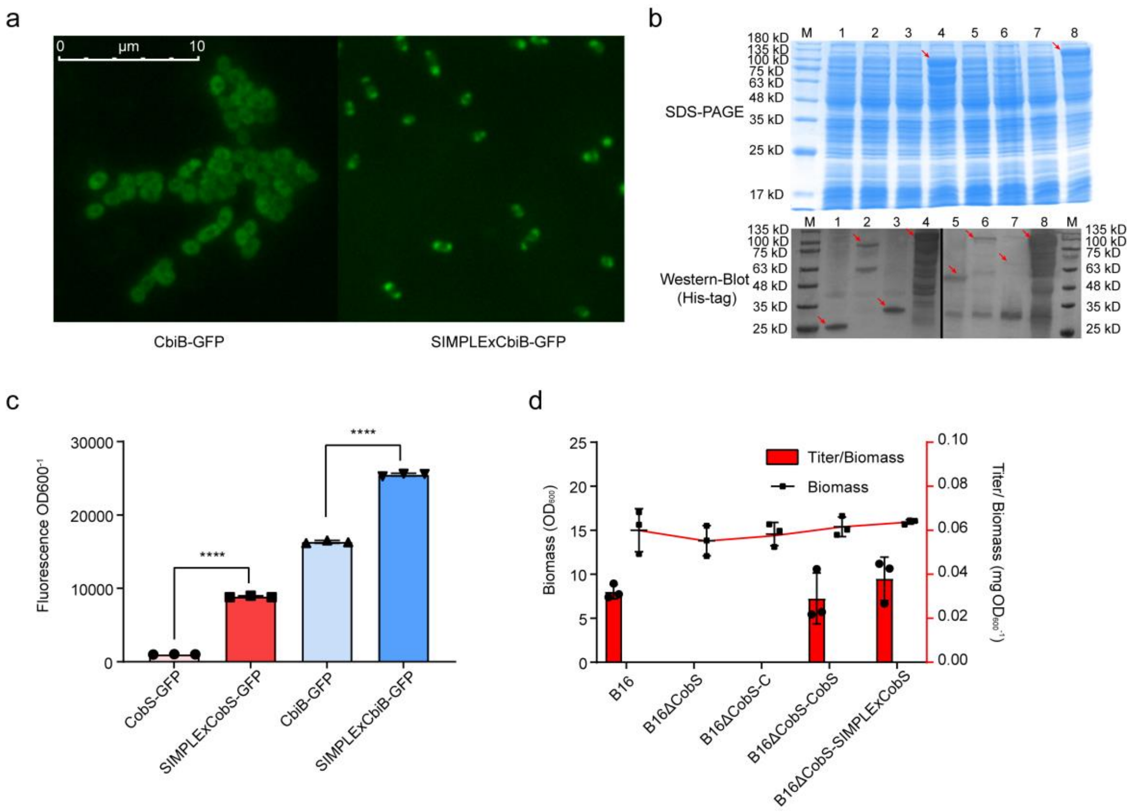

Supplementary Figure 9. Optimization of the recombinant membrane enzymes expression.

a. Fluorescence location of recombinant CbiB and SIMPLExCbiB tagged with GFP in *E. coli* BL21 (DE3) cells detected by fluorescence microscopy. b. SDS-PAGE and western blot analysis of the different forms of recombinant CobS and CbiB. M, Pre-stained protein ladder. 1, CobS. 2, SIMPLExCobS. 3, CbiB. 4, SIMPLExCbiB. 5, CobS-GFP. 6, SIMPLExCobS-GFP. 7, CbiB-GFP. 8, SIMPLExCbiB-GFP. Target proteins were emphasized by red arrows. Antibodies used in western blot analysis: Mouse Anti-His mAb (Catalog # MA1-21315), Peroxidase-Conjugated Goat Anti-Mouse IgG (H+L) (Catalog # 31430). All antibodies were purchased from ZSGB-BIO, China. c. Fluorescence intensity detection of recombinant CobS-GFP, SIMPLExCobS-GFP, CbiB-GFP and SIMPLExCbiB-GFP fusion proteins in recombined *E. coli* BL21 (DE3). All data are shown from n=3 independent biological samples with mean values ± SD. Two-sided unpaired t test is carried out with the fluorescence between original protein expression and SIMPLEx-complex protein expression. Unpaired t test: CobS-GFP to SIMPLExCobS-GFP, P <0.0001 (t=114.4); PfCbiB to SIMPLExPfCbiB,

$P<0.0001$ ($t=59.44$). ****, $P < 0.0001$

d. Enzyme activity of recombinant SIMPLExCobS *in vivo*. B16, recombinant AdoCbl producing strain without plasmid constructed based on *E. coli* ME1655 (DE3). B16ΔCobS, B16 strain with knockout of *cobS* (encoding adenosylcobinamide-5-P synthetase CobS). B16ΔCobS-P, B16ΔCobS with pCDFDuet-1 plasmid. B16ΔCobS-CobS, B16ΔCobS with pCDFDuet-1-CobS. B16ΔCobS-SIMPLExCobS, B16ΔCobS-CobS with pCDFDuet-1-SIMPLExCobS. The titer of vitamin B_{12} was detected by HPLC. All data are shown from n=3 independent biological samples with mean value ± SD. Source data are provided as a Source Data file.

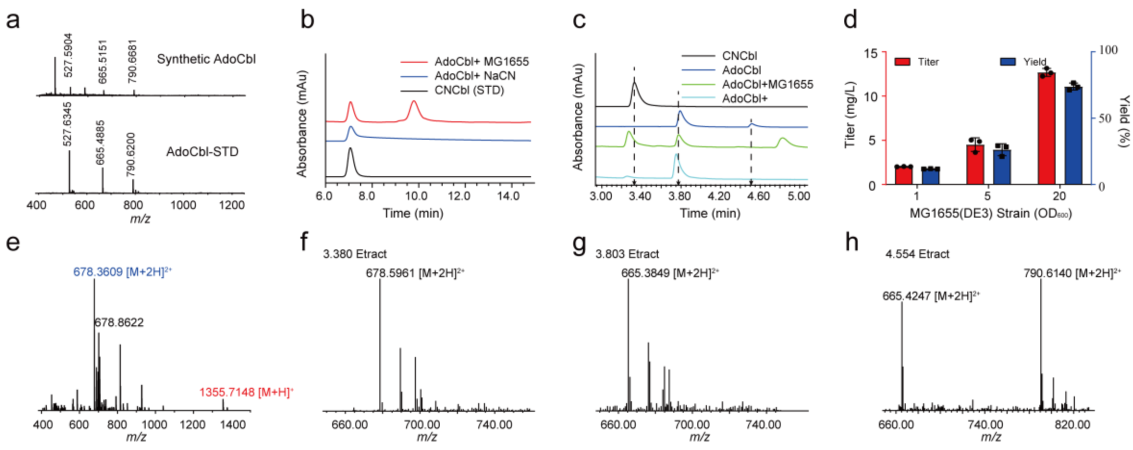

Supplementary Figure 10. Biochemical transformation of AdoCbl standards.

a. direct detection of cell-free synthetic AdoCbl by UPLC-MS. The synthetic AdoCbl and AdoCbl standards (AdoCbl-STD 10 mg/L) are both identified showing in the form of $[M+2H]^{2+}$ (*m/z* is 790.6), OHCbl after C-Co bond broken (*m/z* is 665.5), and MS signal fragment (*m/z* is 527.6). b, HPLC analysis of two methods to transform AdoCbl into CNCbl. AdoCbl+MG1655, AdoCbl was treated with 10% v/v sodium nitrite (8% w/v), 10% v/v acetic acid and appropriate (OD_{600}=20) *E. coli* MG1655 (DE3) wet cells, followed by boiling in a water bath for 30 min. AdoCbl+ NaCN, AdoCbl was treated with 10% v/v sodium cyanide (1% w/v), and irradiated with 270 lux of light at 455 nm for 30min. CNCbl (STD), CNCbl standards. AdoCbl, AdoCbl standards. c. UPLC-MS detection of biochemical transformation of AdoCbl. CNCbl, CNCbl standards. AdoCbl, AdoCbl standards. AdoCbl+MG1655, AdoCbl was treated with 10% v/v sodium nitrite (8% w/v), 10% v/v acetic acid and appropriate (OD_{600}=20) wild type *E. coli* MG1655 (DE3) wet cells, followed by boiling in a water bath for 30 min. AdoCbl+, AdoCbl was treated with 10% v/v sodium nitrite (8% w/v) and 10% v/v acetic acid but without *E. coli* cells, followed by boiling in a water bath for 30 min. d. Biochemical transformation of AdoCbl is related to the quantity of cells. 20 mg/L AdoCbl was treated with 10% v/v sodium nitrite (8% w/v), 10% v/v acetic acid and different quantity of *E. coli* MG1655 (DE3) wet cells, produced CNCbl was detected by HPLC. Reactions were performed in triplicate (n=3 biologically independent samples) and data are presented as mean values ± SD. e, LC-MS detection of CNCbl. CNCbl was easily positive charged with two H^+ (navy word), and traces of CNCbl were positively charged with one H^+ (red

word) in the MS spectrum. f, MS signal extracted from subgraph c at 3.380 min. The mass charge (*m/z*) ratio of 678.5961 is consistent with CNCbl as $[M+2H]^{2+}$ form; g, MS signal extracted from subgraph c at 3.803 min. The mass charge (*m/z*) ratio of 665.3849 is consistent with OHCbl as $[M+2H]^{2+}$ form; h, MS signal extracted from subgraph c at 4.554 min. The mass charge (*m/z*) ratio of 790.6140 is consistent with AdoCbl as $[M+2H]^{2+}$ form; 665.4247 is consistent with OHCbl as $[M+2H]^{2+}$ form. Source data are provided as a Source Data file.

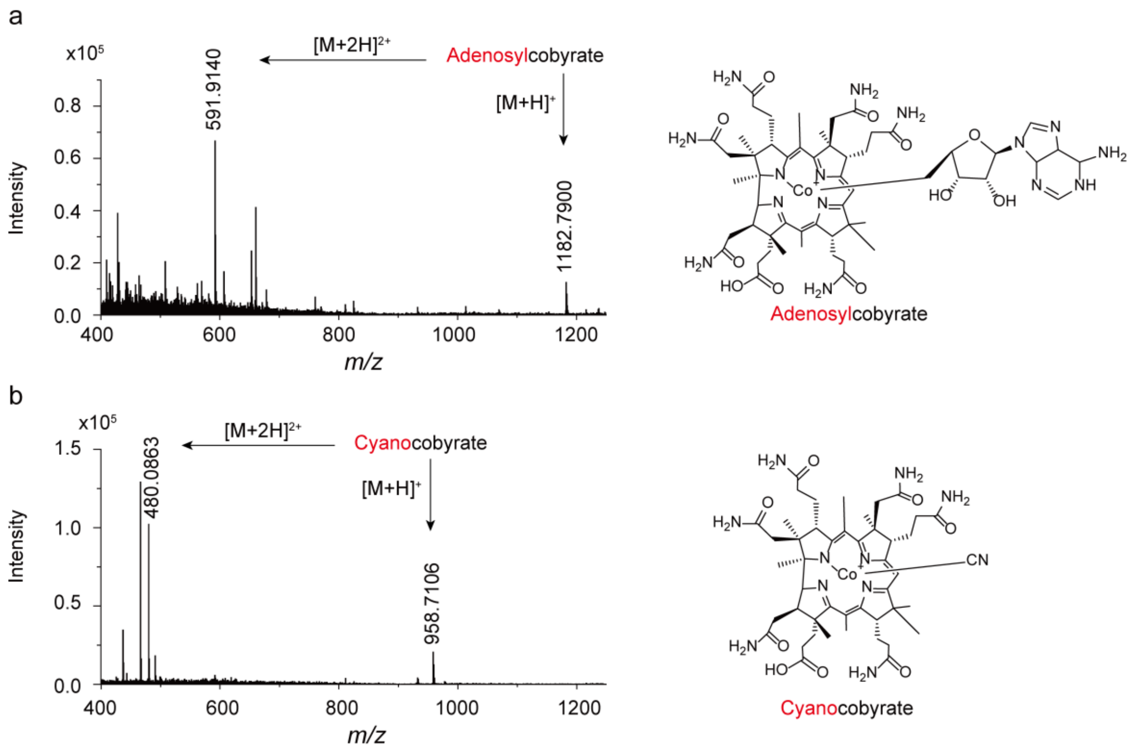

Supplementary Figure 11. biochemical transformation of AdoCby to CNCby.

a, UPLC-MS detection of AdoCby. The AdoCby is easily positive charged with two H^+ (*m/z*, 591.9140), and traces of AdoCby were positively charged with one H^+ (*m/z*, 1182.7900) in the MS spectrum. b, UPLC-MS detection of transformed CNCby. AdoCby samples was treated with 10% v/v sodium nitrite (8% w/v), 10% v/v acetic acid and appropriate (OD_{600}=20) wild type *E. coli* MG1655 (DE3) wet cells, followed by boiling in a water bath for 30 min. The CNCby is easily positive charged with two H^+ (*m/z*, 480.0863), and traces of AdoCby were positively charged with one H^+ (*m/z*, 958.7106) in the MS spectrum. Source data are provided as a Source Data file.

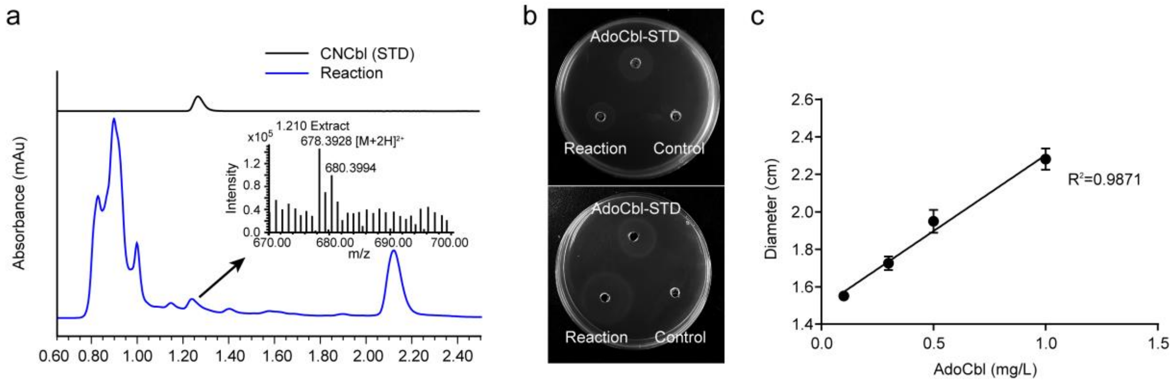

Supplementary Figure 12. Detection of synthetic AdoCbl and confirmation of its bioactivity.

a. UPLC-MS detection of synthetic AdoCbl produced from 5-ALA *in vitro*. The reaction mixture was treated with 10% v/v sodium nitrite (8% w/v), 10% v/v acetic acid and appropriate (OD_{600}=20) *E. coli* MG1655 (DE3) wet cells, followed by boiling in a water bath for 30 min. Samples were analyzed using UPLC-MS and compared with CNCbl standards. The elution gradient was as follows: 30% B (0-5 min), 30-100% B (5-6 min), 100% B (6-7 min), 100-30% B (7-7.5 min), 30% B (7.5-10 min). The extracted MS signal of the sample from the cell-free reaction mixture at 1.210 min is shown in the inner frame. b. Bioassay detection of synthetic AdoCbl produced from HBA. Every sample pool in the plate with *S. typhimurium* AR2680 contained a 20 µl sample or standards in ten-fold (upper) and two-fold (lower) dilution. The whole assay was conducted in the dark. c. The standard curve of S. typhimurium AR2680 in Diameter-Concentration $_{(AdoCbl)}$. Reactions were performed in sextuplicate (n=6 biologically independent samples) and data are presented as mean values ± SD. Source data are provided as a Source Data file.

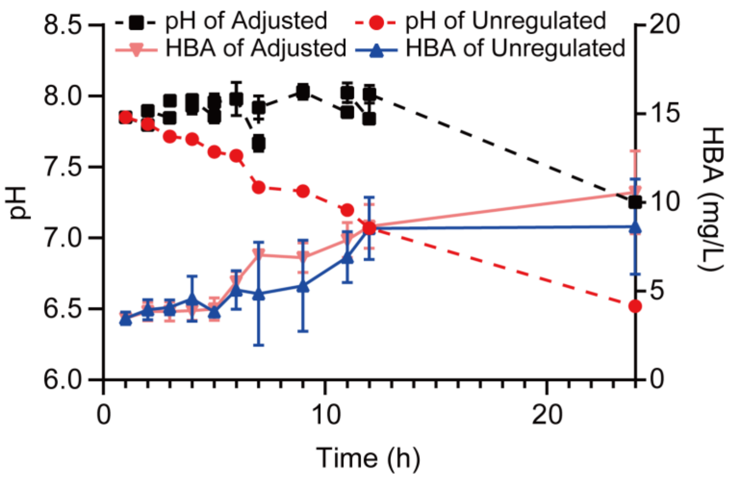

Supplementary Figure 13. pH tracing in HBA synthesis and influence of controlling pH in HBA synthesis.

Unregulated reaction (Unregulated): 0.1 μM HemB, 1 μM HemC, 1 μM HemD, 10 μM CobA, 5 μM MetK, 5 μM PpK, 10 μM MtnN, 2 mM AMP, 1 mM SMPP, 5 mM 5-ALA, 1 mM L-Met, 0.236 mM NADH, 0.236 mM NADPH, 5 mM $MgCl_2$, 10 mM KCl, 5 mM NaCl, 155.8 mg/ml wet cell weight of HBA CCE (corresponding 17.81 mg/ml dry cell weight, 6.23 mg/ml total protein, and 10 OD_{600} HBA cell culture fluid per volume reactant, detail information was illustrated in Supplementary Figure 11) were incubated at 32°C for 12 h. Adjusted reaction (Adjusted): reaction composition is same as unregulated reaction, but was adjusted pH value using 10% NaOH to 7.8-8.0 every hour. The pH value and HBA titer were traced every hour in early 12 h, and final detected in the end of 24 h reaction period. Reactions were performed in triplicate (n=3 biologically independent samples) and shown with line through the mean values ± SD. Source data are provided as a Source Data file.

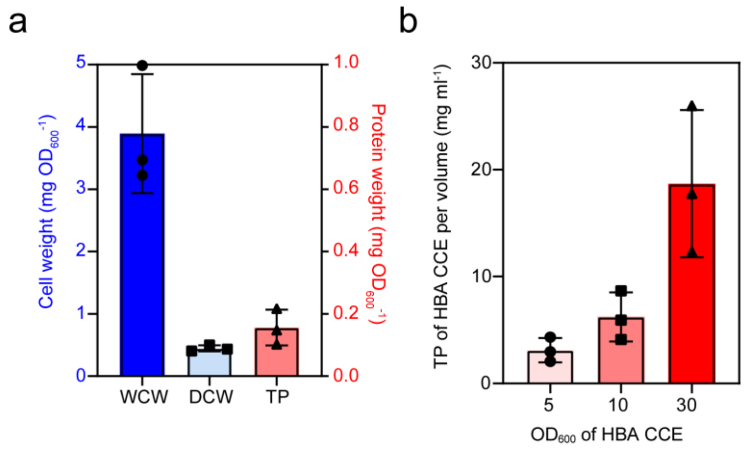

Supplementary Figure 14. Quantification of HBA crude cell extract.

a. quality relation between OD_{600} and Wet Cell Weight (WCW), Dry cell Weight (DCW) and Total Protein (TP). Reactions were performed in triplicate (n=3 biologically independent samples) and data are presented as mean values ± SD. Source data are provided as a Source Data file. b. corresponding TP of HBA CCE in different total OD_{600} quantified used in Supplementary Table 2. Reactions were performed in triplicate (n=3 biologically independent samples) and data are presented as mean values ± SD. Source data are provided as a Source Data file.

Source Data file: Supplementary Figure 9b

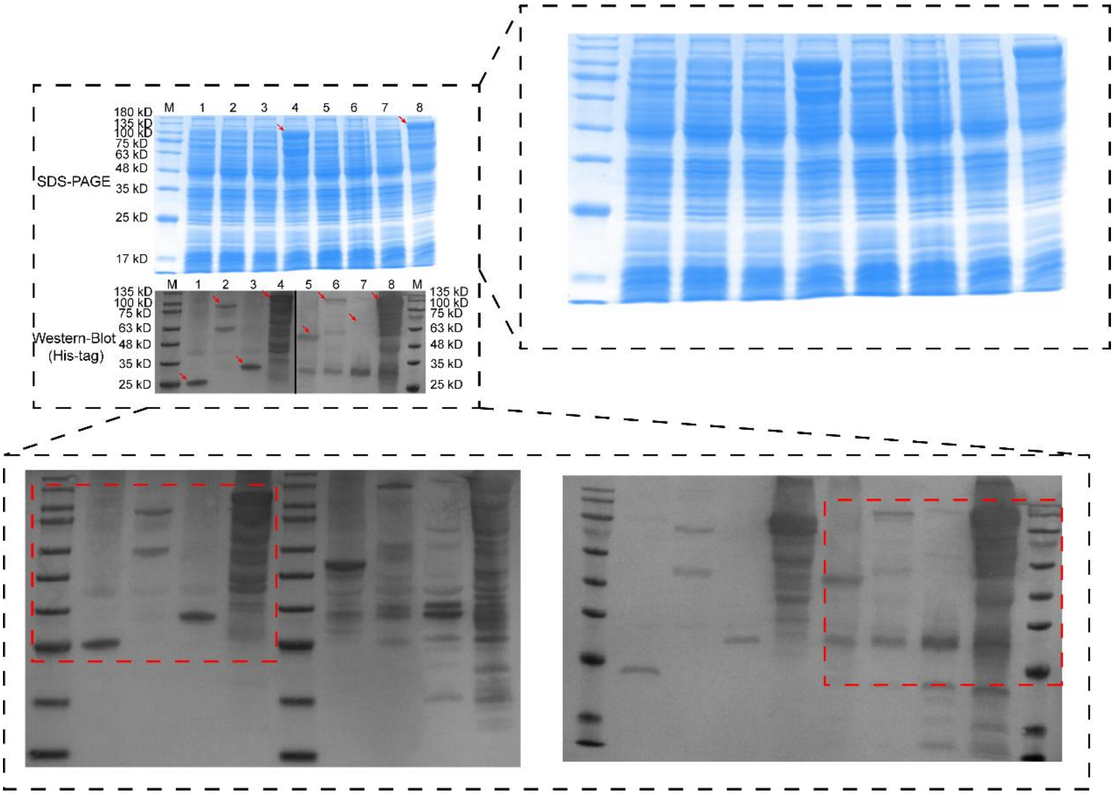

Supplementary References

[1] Consortium, T. U. (2020) UniProt: the universal protein knowledgebase in 2021, *Nucleic Acids Res.* **49 (D1)**, D480-D489 (2021)

[2] Varadi, M. et al. AlphaFold Protein Structure Database: massively expanding the structural coverage of protein-sequence space with high-accuracy models, *Nucleic Acids Res*. **50 (1)**, D439-D444 (2022)

[3] Jumper, J. et al. Highly accurate protein structure prediction with AlphaFold, *Nature*. **596 (7873)**, 583-589 (2021)

[4] Robert, X., Gouet, P. (2014) Deciphering key features in protein structures with the new ENDscript server, *Nucleic Acids Res*. **42**, W320-W324 (2014)
